# A quasi-randomized clinical trial: virtual reality versus proprioceptive neuromuscular facilitation for postmastectomy lymphedema

**DOI:** 10.1186/s43046-020-00041-5

**Published:** 2020-06-15

**Authors:** Doaa Atef, Mohmed Maher Elkeblawy, Ashraf El-Sebaie, Walid Ahmed Ibrahim Abouelnaga

**Affiliations:** 1 Faculty of Physical Therapy, Cairo University, Giza, Egypt; 2 National Center of Research, Giza, Egypt; 3 Faculty of Medicine, Cairo University, Cairo, Egypt

**Keywords:** Virtual reality, Proprioceptive neuromuscular facilitation, Postmastectomy lymphedema, Circumferential measurement, QuickDASH-9 scale

## Abstract

**Background:**

Postmastectomy lymphedema can be considered the main cause of upper extremity functional impairment in patients with breast cancer. Fatigue, pain, and limited range of motion are common symptoms. If left untreated, lymphedema causes cellulitis, which can lead to gangrene in rare cases. This study was carried out to identify and compare the therapeutic advantages of virtual reality-based exercises and proprioceptive neuromuscular facilitation for postmastectomy lymphedema. Thus, a quasi-randomized comparative study of thirty female patients with unilateral postmastectomy lymphedema was conducted. Fifteen patients performed virtual reality-based exercises as well as manual lymphatic drainage, pneumatic compression, and home programs, while the other fifteen patients performed proprioceptive neuromuscular facilitation as well as manual lymphatic drainage, pneumatic compression, and home programs. The excess arm volume between the healthy and affected limbs was estimated before and after eight sessions of treatment for both groups. In addition, the affected limb functional score was calculated. Arm volume was calculated by the truncated cone formula and girth measurements obtained by the circumferential method. The Arabic version of the QuickDASH-9 scale was used to assess extremity function.

**Results:**

The excess arm volume significantly decreased in both the virtual reality group (*p* = 0.001) and proprioceptive neuromuscular facilitation group (*p* = 0.005), and there was no significant difference between the two groups (*p* = 0.902). Age was inversely related to the improvement percentage of the QuickDASH-9 score in the virtual reality group. The functional improvement percentage was statistically significantly different between the two groups (*p* = 0.045).

**Conclusion:**

It can be concluded that both virtual reality and proprioceptive neuromuscular facilitation have a beneficial therapeutic effect on edema in patients with unilateral postmastectomy lymphedema; neither method was found to be superior, except virtual reality was found to be superior to proprioceptive neuromuscular facilitation in motivating patients and providing visual feedback.

**Trial registration:**

ClinicalTrials.gov, NCT04185181 Registered 4 December 2019 - Retrospectively registered.

## Background

Upper limb lymphedema is the most common cause of morbidity after mastectomies with axillary lymph node removal. This condition is characterized by the abnormal accumulation of fluids and proteins in the intercellular space, chronic inflammation, and edema [1].

The lymphatic system involves a negative pressure system that is supported by valves, and muscles contract to generate pressure in the surrounding tissue, as in the lower leg veins [2]. Furthermore, mechanical obstruction caused by surgical procedures or other kinds of lymphatic compromise can create outflow resistance, which consequently increases the lymphatic pressure. The increased pressure can impair the lymphatic valve. Valvular failure can result in lymph backflow across the skin [3]. Seventy-five percent of patients manifest swelling within 3 years after the surgery. Afterwards, the risk of developing lymphedema increases by 1% annually [4]. Infection can be considered a causative factor of lymphedema as well as a complicating factor for previously existing lymphedema [5].

The most common complaint of patients with postmastectomy lymphedema is upper limb functional impairment. Limitations in range of motion (ROM) after the treatment of breast cancer can be due to the formation of scar tissue at the incisional site, fibrosis induced by radiation, protective forward shoulder posturing, pain, and/or disuse. Limited shoulder abduction may restrict daily activities, such as reaching behind the head when combing or washing one’s hair or movements that require maximum shoulder elevation, such as reaching objects located high on a shelf [6].

Virtual reality (VR) systems are considered good training tools because they encourage a high level of training targeting specific deficits and assist patients in training on task-oriented activities. In addition, VR systems permit timed monitoring and can be used to quantitatively assess individuals’ impairments (regarding the relevant properties), performance, and recovery. Additionally, VR environments attract patients’ attention, motivate them to practice exercises, and provide patients with a sense of achievement [7, 8]. Lymphedema patients can perform arm function training with different games related to daily life activities [9].

A continuous condition of edema and chronic inflammation due to abnormal tissue protein accumulation, which is caused by lymphedema, leads to a loss of muscle flexibility and limitations in joint movements [10]. Therefore, proper exercise is important to maintain optimal function [11].

Proprioceptive neuromuscular facilitation (PNF) stretching can be defined as the act of moving within a range without pain [12], and it has a significant role in preventing and reducing exercise injuries by improving flexibility and increasing blood flow [13]. Moreover, PNF stretching folds exercise accuracy and muscle activity in addition to improving body coordination [14]. PNF stretching has been shown to considerably decrease the edema rate [10]. After PNF D2 flexion and breathing exercises, parameters of the lymphedematous upper extremity and the body water volume reduced in a previous study [15].

Manual lymphatic drainage (MLD) is a specific manual technique that is frequently performed as a part of complete decongestive therapy. It involves making gentle strokes in the direction of lymphatic flow, i.e., in the direction from the distal segment to the proximal segment. The main goal of lymphatic drainage is to stimulate lymphokinetic activity and activate the functional units of lymphatic vessels. It lasts for 30 to 45 min or longer. Traditionally, it starts with manual pressure on the central lymph nodes (e.g., those on the neck, abdomen), intact lymph nodes in nearby areas (e.g., the axilla, groin), and areas of anastomosis (e.g., the thorax, lateral trunk), followed by MLD of the limb from proximal to distal segments. The strokes should never cause pain or be unpleasant for the patient. MLD is recommended before compression therapy. It is not considered a stand-alone treatment [16].

Pneumatic compression devices are used in the management of lymphedema. They work by promoting venous and lymphatic return from distal segments of the body back to proximal areas. The simplest device has a single sleeve that alternately inflates and deflates. More complex devices have multiple sleeves that are programmed to start from the proximal to the distal segments or vice versa. Another device uses an algorithm that was designed to mimic MLD [17].

This study was important to conduct due to the lack of data and knowledge in the physical therapy field about the effectiveness of VR and PNF in reducing lymphedema and improving function in postmastectomy patients. The objective of this study was to compare the therapeutic efficacy of VR and PNF in treating lymphedema and improving function in postmastectomy patients. Lymphedema was assessed by the circumferential method, and function was assessed by the QuickDASH-9 scale.

## Methods

### Study design and data collection

A quasi-randomized comparative study including 30 unilateral postmastectomy lymphedema (UPML) patients was performed. The study was conducted in the Physical Therapy Department at the National Cairo Institute in Egypt from June 2018 to August 2019. The research ethics committee of the Faculty of Physical Therapy at Cairo University approved this study. All included patients signed informed consent forms before the start of data collection.

**Fig. 1:**
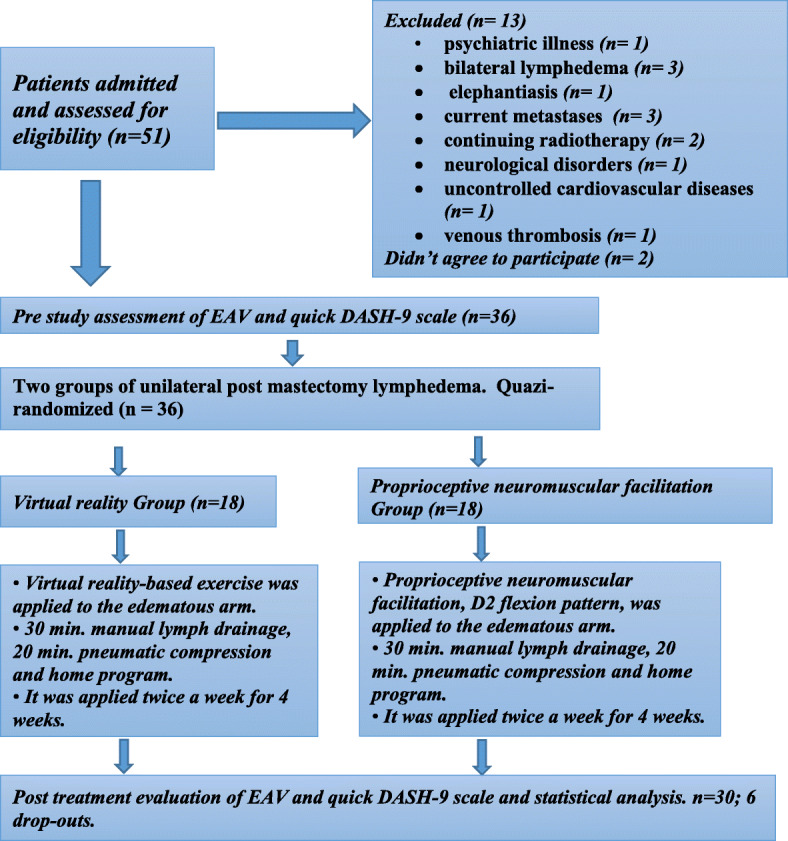
Participants’ inclusion process flowchart showing those with unilateral postmastectomy lymphedema who underwent the QuickDASH-9 assessment, excess arm volume (EAV) measurement, virtual reality-based exercise, and proprioceptive neuromuscular facilitation

The aim of this study was to compare the therapeutic efficiency of VR and PNF in treating lymphedema and improving function in UPML patients. The study design was parallel, and the patients were subdivided into two equal groups, with 15 patients in each. The quasi-sampling method was performed by the alternative allocation of patients in both groups. This was not a blinded study (Fig. 1).

Only female patients were included. Their age range was from 40 to 65 years. The patients had UPML. All patients had undergone modified radical mastectomy with axillary lymph node dissection. The postsurgical duration was at least 6 months. The inter-limb volume difference was at least 5%, which was calculated by the truncated cone formula. The patients were categorized as having stage I or stage II lymphedema, according to the International Society of Lymphology classification system. Stage I represents an early accumulation of fluid relatively high in protein content (e.g., in comparison with “venous” edema), which subsides with limb elevation. The pitting phenomenon may occur. An increase in various types of proliferating cells may also be observed. Stage II shows that limb elevation alone rarely reduces tissue swelling, and the pitting phenomenon is obvious. Later, in stage II, the limb may not pit as excess subcutaneous fat, and fibrosis may develop. At each stage, the severity can be determined by the difference in the limb volume between the affected and unaffected limb as follows: minimal (a 5 to 10% increase in the volume), mild (a 10 to 20% increase in the volume), moderate (a 20 to 40% increase in the volume), or severe (a 40% or higher increase in the volume) [18]. Patients with musculoskeletal or neurological disorders that can impair performance during training and testing, visual disorders that can affect video game-based exercise performance, uncontrolled cardiovascular or pulmonary diseases, psychiatric illness, bilateral lymphedema, elephantiasis, current metastases, continuing radiotherapy, or venous thrombosis were excluded. The included patients did not undergo physical therapy before the current treatment, or the last time they participated in therapy sessions was at least 3 months ago.

The patients in the VR group were asked to freely move the shoulder joint. After this warm-up, the patients practiced a 30-min exercise program using a Nintendo Wii® video game. During the sessions, the patients stopped for 1 min between games [19]. Tennis, triceps extension, and rhythmic boxing were the games used. The patients participated in 2 sessions per week for 4 weeks.

For the PNF group, the starting position for the exercise was in the supine position, with the shoulder joint extended, adducted, and internally rotated; the elbow extended; the forearm pronated; the wrist flexed; and the fingers flexed. The patients slowly stretched their shoulders by flexing, abducting, and externally rotating them; extending their elbows; and supinating their forearm. They slowly stretched their wrist and fingers by extending them and pointing their thumbs towards the floor. This stretch was combined with inspiration followed by holding the breath at the end position for 5 s. Afterwards, while expiring, the patients slowly returned their arms to the starting position. This exercise was performed 10 times, followed by 1 min of rest, and repeated for 30 min [15]. The patients participated in 2 sessions per week for 4 weeks.

Both groups underwent 20 min of pneumatic compression at a pressure of 60 mmHg twice a week for 4 weeks. A multi-sleeve device was used [20]. In addition to 30 min of MLD twice a week for 4 weeks, a home program including elevation, positioning, exercises, and skincare advice was performed. Multi-layer bandaging was not applied due to patient incompliance. Educational instructions were given to the patients in printed handouts. These instructions regarded the lymphatic system and its function, precautions (e.g., avoiding subjecting the affected limb to heat), and figures showing the home program exercises.

In the assessment, the circumferential method and the truncated cone formula were used to calculate the volumes, and the Arabic version of the QuickDASH-9 scale was used to assess upper limb function.

### Circumferential measurements

Circumferential measurements depend on specified distances along the length of the limb, but the hand volume cannot be calculated. This method provides information on the localization of the swelling [16].

In the current study, the circumference of the upper limb was measured every 4 cm from the wrist and to the axilla, and the volume was calculated with the truncated cone formula (as the limb is viewed as a series of truncated cones or frustum-shaped segments). The volume of each segment = *L*/12π (C1^2^ + C1 C2 + C2^2^), where C1 and C2 are the circumferences of the ends of a segment length (*L*). The total limb volume was defined as the sum of the segment volumes [21]. The excessive volume of the UPML was calculated by subtracting the volume of the non-affected upper limb from that of the lymphedematous upper limb. The excess arm volume (EAV) = VL − VH, where VL refers to the lymphedematous limb’s volume, and VH refers to the healthy extremity’s volume. The EAV was measured before and after treatment. These measurements were taken before treatment and after 4 weeks of treatment for both groups. Then, the percent improvement in the EAV was calculated by the equation [(pre EAV − post EAV) × 100/pre EAV] to compare the magnitude of improvement between the two groups [22].

### QuickDASH-9 scale

The affected upper limb function was assessed using the validated Arabic version of the QuickDASH-9 scale before the treatment and after 4 weeks of treatment for both groups. It includes 9 items, and each item in the QuickDASH-9 is scored on a 4-point Likert scale (0–4); a higher value corresponds to greater disability/severity of symptoms and reduced function. A scaled score can be derived even if one item is missed. The total scores are converted into a scaled score (0–100) using a formula. The Arabic QuickDASH-9 score = [(sum) × 1.1] × 5/2, and a missing response is replaced by the average of the remaining scores [23]. Then, the percent improvement in the QuickDASH-9 score was calculated by the following equation: [(pre-treatment score − post-treatment score) × 100/pre-treatment score].

### Statistical methods

The data were processed and analyzed using the statistical package for the Social Sciences (SPSS), version 25 (IBM Corp., Armonk, NY, USA). The data are summarized as follows:

1. Means ± standard deviations (SD) for data with normal distribution {the age, weight, height, and body mass index (BMI)}.
2. Median (interquartile range [IQR]) for data with non-normal distribution {number of dissected lymph nodes, QuickDASH-9 scores, and EAV}.
3. Frequencies (number of cases) and relative frequencies (percentages) for nominal variables {handedness, menopause status, and stage of lymphedema}.

The non-parametric Mann-Whitney *U* test and non-parametric Wilcoxon signed-rank test were used to compare the EAV and the QuickDASH-9 scores between groups and between the pre- and post-intervention time points within each group, respectively. The Shapiro–Wilk test was used to assess normality [24]. The correlations between quantitative variables were assessed using the Spearman correlation coefficient [25]. The statistical significance level was set to be 0.05.

## Results

This study included thirty females. Their ages ranged between 40 and 65 years, and the mean ages were 54.07 ± 8.28 and 53.07 ± 7.24 years for the VR and PNF groups, respectively (Table 1).

**Table 1:** Demographic data for the quantitative and qualitative variables

**Quantitative variables**	**VR group**		**PNF group**		***p***
	**Mean ± SD [or median (IQR)]**		**Mean ± SD [or median (IQR)]**		
Age (years)	54.07 ± 8.28		53.07 ± 7.24		0.727
Weight (kg)	88.83 ± 17.73		94.13 ± 12.38		0.351
Height (cm)	160.60 ± 3.74		162.33 ± 3.75		0.216
BMI (kg/m^2^)	34.56 ± 7.65		35.73 ± 4.71		0.617
No. of LN*	17 (6)		18 (9)		0.653
**Qualitative variables**	**Count**	**Percentage (%)**	**Count**	**Percentage (%)**	***p***
Dominant hand	8	53.3	11	73.3	0.256
Non-dominant hand	7	46.7	4	26.7	
Pre menopause	4	26.7	4	26.7	1
Post menopause	11	73.3	11	73.3	
Stage I lymphedema	11	73.3	8	53.3	0.256
Stage II lymphedema	4	26.7	7	46.7	

*Number of dissected lymph nodes

There were no statistically significant differences in the ages, weights, heights, body mass indexes, and numbers of dissected lymph nodes between the two groups (Table 1). Additionally, there were no statistically significant differences in the handedness, menopausal status (pre- or post-menopausal), or stage of lymphedema (stage I or stage II) between the two groups (Table 1).

There was a statistically significant difference in the EAVs (*p* = 0.001) and QuickDASH-9 scores (*p* = 0.001) in the virtual reality group from before to after the intervention. Additionally, a significant difference in the EAVs (*p* = 0.005) and QuickDASH-9 scores (*p* = 0.003) was found in the PNF group (Table 2). The differences in EAVs and QuickDASH-9 scores between the two groups were not significant (Table 2).

**Table 2:** Comparison between the pre- and post-treatment median [IQR] values of all the measured variables within each group and between the two groups

	**VR group**				**PNF group**				***p***
**EAV (ml)**									
	Median (IQR)	Q1	Q3	*p*	Median (IQR)	Q1	Q3	*p*	
**Pre**	9655.27 (15511.84)	3624.49	19136.33	0.001	10645.92 (11986.67)	3822.38	15809.05	0.005	0.744
**Post**	6854.23 (9459.49)	2728.47	12187.96		5718.9 (6834.54)	4412.98	11247.52		0.902
**QuickDASH-9 score**									
	Median (IQR)	Q1	Q3	*p*	Median (IQR)	Q1	Q3	*p*	
**Pre**	55 (30.25)	41.25	71.5	0.001	49.5 (22)	38.5	60.5	0.003	0.539
**Post**	38.5 (27.5)	24.75	52.25		38.5 (19.25)	30.25	49.5		0.935

**Fig. 2:**
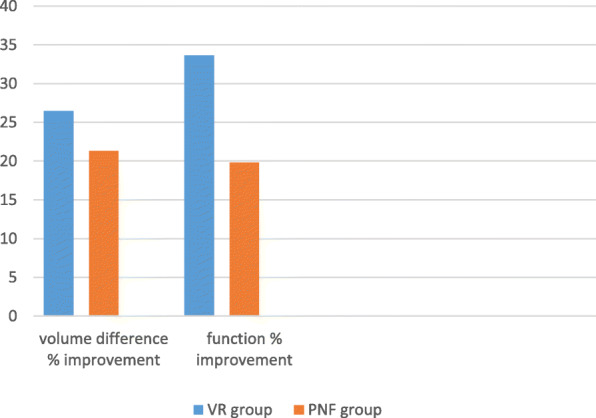
The percentage improvement in all the measured variables between the two groups

The analysis of the improvement percentage showed that the lymphedema state and upper limb function improved more in the virtual reality group than in the PNF group (Fig. 2). The mean percentage improvement values in the EAV were 26.47 ± 23.59 and 21.33 ± 37.46 for the VR group and PNF group, respectively. The difference between the groups was not statistically significant (*p* = 1). The mean percentage improvement values in the QuickDASH-9 score were 33.66 ± 16.87 and 19.81 ± 20.14 for the VR group and the PNF group, respectively. The difference between groups was statistically significant (*p* = 0.045).

There were no relationships between the EAV percentage improvement and patient age (*p* = 0.78 and 0.727 in the VR and PNF groups, respectively), BMI (*p* = 0.351 and 0.344 in the VR and PNF groups, respectively), or the number of dissected lymph nodes (*p* = 0.734 and 0.115 in the VR and PNF groups, respectively) in either group. There was an inverse relationship between age and the QuickDASH-9 score percentage improvement in the VR group (*r* = − 0.548, *p* = 0.034). There were no relationships between the QuickDASH-9 score percentage improvement and patient age (*p* = 0.970 in the PNF group), BMI (*p* = 0.082, 0.331 in the VR and PNF groups, respectively), or number of dissected lymph nodes (*p* = 0.317, 0.677 in the VR and PNF groups, respectively) (Table 3).

**Table 3:** Relationships between the percentage EAV improvement and percentage functional improvement and patient age, BMI, and number of dissected lymph nodes

	**EAV percentage improvement**				**QuickDASH-9 score percentage improvement**			
	VR group		PNF group		VR group		PNF group	
	Correlation coefficient	*p* value	Correlation coefficient	*p* value	Correlation coefficient	*p* value	Correlation coefficient	*p* value
Age	0.079	0.780	0.098	0.727	− 0.548	0.034	0.011	0.970
BMI	0.259	0.351	0.263	0.344	− 0.463	0.082	0.270	0.331
LN no.*	0.096	0.734	− 0.424	0.115	− 0.277	0.317	− 0.117	0.677

*Number of dissected lymph nodes

There were no relationships between the EAV percentage improvement and patient handedness (*p* = 0.694, 0.753 in the VR and PNF groups, respectively), menopausal status (*p* = 0.177, 0.949 in the VR and PNF groups, respectively), or stage of lymphedema (*p* = 0.177, 0.189 in the VR and PNF groups, respectively) in either group. There were no relationships between the QuickDASH-9 score percentage improvement and patient handedness (*p* = 0.779, 0.177 in the VR and PNF groups, respectively), menopausal status (*p* = 0.571, 0.851 in the VR and PNF groups, respectively), and stage of lymphedema (*p* = 0.949, 0.336 in the VR and PNF groups, respectively) in either group (Table 4).

**Table 4:** Relationship between the EAV percentage improvement and percentage functional improvement and patient handedness, menopausal status, and stage of lymphedema

	**EAV percentage improvement**					
	**VR group**			**PNF group**		
	Mean ± SD		*p* value	Mean ± SD		*p* value
Handedness	**Yes**	**No**		**Yes**	**No**	
	22.76 ± 20.79	30.70 ± 27.47	0.694	28.17 ± 23.02	2.52 ± 64.33	0.753
Menopause	**Pre**	**Post**		**Pre**	**Post**	
	10.78 ± 12.66	32.17 ± 24.43	0.177	7.25 ± 68.36	26.45 ± 21.31	0.949
Stage of lymphedema	**Stage I**	**Stage II**		**Stage I**	**Stage II**	
	31.64 ± 25.15	12.24 ± 10.94	0.177	24.06 ± 49.80	18.21 ± 18.97	0.189
	**QuickDASH-9 score percentage improvement**					
	Group A			Group B		
	Mean ± SD		*p* value	Mean ± SD		*p* value
Handedness	**Yes**	**No**		**Yes**	**No**	
	31.46 ± 9.78	36.16 ± 23.20	0.779	14.95 ± 17.43	33.19 ± 23.54	0.177
Menopause	**Pre**	**Post**		**Pre**	**Post**	
	37.12 ± 13.81	32.39 ± 18.29	0.571	22.45 ± 18.86	18.86 ± 21.38	0.851
Stage of lymphedema	**Stage I**	**Stage II**		**Stage I**	**Stage II**	
	33.96 ± 17.98	32.83 ± 15.78	0.949	25.29 ± 18.47	13.56 ± 21.51	0.336

The percentage improvement in the EAV was significantly correlated with the percentage improvement in the affected upper limb function in the PNF group, with *r* = 0.554 and *p* = 0.032. There was no significant correlation in the VR group (*r* = − 0.182, *p* = 0.516).

### Power calculation

A post hoc power calculation of sample size was carried out using the G*POWER statistical software (version 3.1.9.2; Franz Faul, Universitat Kiel, Germany). The modified QuickDASH-9 score percentage improvement was the primary outcome of interest. Results revealed that the power = 0.544. The low power value can be attributed to the small sample size.

## Discussion

There is a lack of data and knowledge in the physical therapy field about the effectiveness of VR and PNF in reducing lymphedema and improving function in postmastectomy patients; thus, the aim of the current study was to assess and compare the efficacy of VR and PNF in treating UPML.

Lymphedema may lead to severe consequences related to patients’ functional and psychological aspects of life, reducing quality of life. Functional impairment can result from pain, heaviness, and decreased ROM of the diseased limb or by infection and impaired wound healing [16].

There are two types of lymphedema prevention, which are termed primary and secondary prevention. Primary prevention is designed for all patients at risk of developing lymphedema. Early management of lymphedema is implemented to prevent the occurrence of the disorder. A preoperative plan involving evaluation and prevention education should be developed for all patients undergoing treatment involving a risk of developing lymphedema [26]. Secondary prevention targets the prevention of lymphedema complications. Perfect skincare, as well as compliance in putting on pressure garments and self-management, is definitive in preventing the progression of edema and development of dystrophic complications [16].

Both groups in the current study had similar demographic and clinical characteristics. There were no significant differences between groups. Both groups showed significant improvement in the EAV, which was calculated by the truncated cone formula and measurements of the arm circumference taken before the first session and after the last session (after 4 weeks). Similarly, upper limb function improved in both groups. Both VR and PNF were found to be successful after 4 weeks without significant differences between the two treatments.

Regarding the improvement in the VR group, the protocol for VR-based exercises for the upper limb on the same side of the mastectomy suggested to be efficient in improving upper limb function and decreasing the risk of anxiety and depression [19]. The VR system is a useful tool in physical rehabilitation since it facilitates a training exercise session that is tailored to the patient’s needs and has an engaging environment that attracts attention and focus and motivates the patient to perform the exercises. Furthermore, patient performance can be quantified, allowing clinicians to continuously monitor patients during rehabilitation [7, 8]. The magnitude of improvement in the VR group in the current study may be attributed to these presented benefits of the VR system.

The improvement in the EAV was significantly correlated with the improvement in function in the PNF group. However, in the VR group, there was no significant correlation. The results showed that function improved by 33.66%, while the EAV improved by 26.47% in the VR group. The mean pre-treatment QuickDASH-9 score was 54.82, while the meant post-treatment score was 38.50. This change can be attributed to the advantage of VR in motivating and involving patients in interactive games that help the patients perform repetitive and quick arm movements. Thus, the percentage functional improvement was greater than the percentage EAV improvement; in contrast, the PNF group, the magnitudes of improvement in the two variables were significantly correlated. According to Singh et al. [27], the participation of less physically active patients in regular exercise is of greater importance than that of the general population due to the fact that adult patients with disabilities are more prone to secondary complications such as fatigue, pain, and deconditioning. People with disabilities have limited involvement in physical activity, and using technology may promote motivation, adherence, and involvement in physical activity and exercise programs. VR games intended to be played during individuals’ spare time, but Singh et al. found that VR games can be beneficial in rehabilitation in their regional studies. Significant improvements were demonstrated in the hand reaction time, with an approximately 12% reduction in the average reaction time after treatment. This result can be attributed to quick and repetitive arm and hand movements that are practiced in the interactive VR games. The current study shows that VR has large effects on the motivation and involvement of patients to exercise and yields improvements in function. Unfortunately, the hand reaction time or the average time was not measured in the current study.

The improvement in the QuickDASH-9 score was inversely related to age in the VR group. This result can be explained by a decline in the physical ability of older and/or obese patients with postmastectomy lymphedema. Borman et al. found that elderly women with postmastectomy lymphedema have upper extremity impairment, which may subsequently threaten their ability to live independently. While age is not a modifiable factor, BMI is modifiable. Therefore, Borman et al. recommended in their study that clinicians focus on reducing not only the volume of the edematous limb but also the patient’s BMI. In their study, BMI was strongly correlated with the DASH score, and the authors concluded that obese patients have more severe upper extremity functional disability [28]. In our study, the relationship between BMI and improvement in the QuickDASH-9 score was not significant, but it approached significance. VR videogames require interaction, which requires some physical fitness, and they were played from a standing position in front of the display. PNF is an easier exercise and is performed from a supine or sitting position. Therefore, there was no relation between age and QuickDASH-9 score improvement in the PNF group.

It is suggested that clinicians use VR with postmastectomy patients with upper limb functional limitations. Researchers should study the difference between the effect of VR alone and the effect of VR combined with complete decongestive therapy.

For the improvement in the PNF group, the results showed that function improved by 19.81%, while the EAV improved by 21.33%. Moseley et al. [29] analyzed breathing combined with gentle exercises of the arm and observed a reduction in upper extremity lymphedema immediately after exercise and 30 min, 24 h, and a week after exercise. At 1 month after exercise, this parameter decreased double the amount that it improved in the first assessment. A positive effect on lymphedema can be attributed to the combined positive effects of breathing and upper extremity exercises. This combined effect was achieved in the current study by the combination of PNF and breathing exercise. In conclusion, Hwang et al. [10] encouraged the use of both PNF and massage techniques to decrease the rate of edema. After 4 weeks of treatment, edema was reduced to a larger extent in the PNF group than in the massage group. The conclusion of the current study is consistent with that of Hwang et al. regarding the positive effect of PNF on lymphedema. A study by Ha et al. showed that combined MLD and PNF has a powerful synergistic effect on the edema volume in lymphedema patients [30]. The results of a case study by Hwang et al. concluded that the D2 flexion pattern of PNF combined with breathing exercises results in a reduction in the circumference of the right upper limb and a reduction in the volume of water in the body. Furthermore, the restricted ROM in the right upper limb improved [19]. One of the most effective ways of varying tissue pressure is through musculoskeletal movement, such as that created by the PNF. Since there was a reduction in edema in the PNF group, differences in the total tissue pressure may have caused the extent of lymph propulsion and clearance to increase. Pressure differentials created by the diaphragm are also shown to influence lymph flow, helping to propel lymph centrally for drainage into the thoracic lymphatic ducts [29].

It is suggested that clinicians to use PNF combined with breathing exercises, MLD, pneumatic compression, and exercises. Researchers should study the combined effect of PNF and breathing exercises with a large sample size and a long follow-up period.

The limitations of the current study were the absence of a third group receiving complete decongestive therapy (CDT) alone, the small sample size, and the incompliance of patients, which restricted the application of multilayer bandaging. Thus, in future studies, it is suggested that a third group undergoing CDT is included, a randomized sampling method is used, multilayer bandaging for the CDT group is performed, the patients are stratified by their level of literacy (illiterate or literate), the relation between educational level and improvement in outcomes is studied, and a larger sample size is used.

## Conclusion

It can be concluded that VR is beneficial in reducing postmastectomy lymphedema and can be used as an exercise-based technique in these patients, as it motivates and provides visual feedback to patients. Additionally, PNF is beneficial in reducing postmastectomy lymphedema, especially if it is accompanied by a breathing exercise that enhances lymphatic drainage by diaphragmatic movement during abdominal breathing, in addition to promoting relaxation. Both techniques have a nearly similar effect on UPML.

## Data Availability

The dataset supporting the conclusions of this article is included within the article.

## Abbreviations

DASH: Disability of the arm, shoulder, and hand
ROM: Range of motion
VR: Virtual reality
PNF: Proprioceptive neuromuscular facilitation
MLD: Manual lymphatic drainage
UPML: Unilateral postmastectomy lymphedema
EAV: Excessive arm volume
SD: Standard deviation
BMI: Body mass index
IQR: Interquartile range
CDT: Complete decongestive therapy
